# Neoadjuvant chemotherapy followed by fast-track cytoreductive surgery plus short-course hyperthermic intraperitoneal chemotherapy (HIPEC) in advanced ovarian cancer: preliminary results of a promising all-in-one approach

**DOI:** 10.2147/CMAR.S153327

**Published:** 2017-12-13

**Authors:** Thales Paulo Batista, Vandré Cabral G Carneiro, Rodrigo Tancredi, Ana Ligia Bezerra Teles, Levon Badiglian-Filho, Cristiano Souza Leão

**Affiliations:** 1 Department of Surgery/Oncology, IMIP – Instituto de Medicina Integral Professor Fernando Figueira; 2 Department of Surgery, UFPE – Universidade Federal de Pernambuco; 3 Department of Gynecology, HCP – Hospital de Câncer de Pernambuco; 4 Department of Clinical Oncology, IMIP – Instituto de Medicina Integral Professor Fernando Figueira; 5 Department of Clinical Oncology, HCP – Hospital de Câncer de Pernambuco; 6 Department of Anaesthesiology, IMIP – Instituto de Medicina Integral Professor Fernando Figueira, Recife; 7 Department of Gynecology, AC Camargo Cancer Center, Sao Paulo; 8 Department of Surgery, IMIP – Instituto de Medicina Integral Professor Fernando Figueira, Recife, Brazil

**Keywords:** hyperthermia, peritoneal neoplasms, peritoneal surface malignancy, peritoneal carcinomatosis, ovarian neoplasms

## Abstract

**Purpose:**

Hyperthermic intraperitoneal chemotherapy (HIPEC) has been considered a promising treatment option for advanced or recurrent ovarian cancer, but there is no clear evidence based on randomized controlled trials to advocate this approach as a standard therapy. In this study, we aim to present the early outcomes and insights after an interim analysis of a pioneering clinical trial in Brazil.

**Methods:**

This study was a cross-sectional analysis of early data from our ongoing clinical trial – an open-label, double-center, single-arm trial on the safety and efficacy of using HIPEC for advanced ovarian cancer (ClinicalTrials.gov: NCT02249013). A fast-track recovery strategy was also applied to improve patient outcomes.

**Results:**

Nine patients with stage IIIB (n=1) or IIIC (n=8) epithelial malignancies were enrolled until February 2017. The median (range) serum CA125 level at diagnosis was 692 (223.7–6550) U/mL. The median number of preoperative cycles of intravenous (i.v.) chemotherapy was 3 (2–4), resulting in peritoneal cancer index scores of 9 (3–18) at the time of HIPEC. Time of restarting i.v. chemotherapy was 37 (33–50) days with all patients completing 6 cycles as planned. The median operation time was 395 (235–760) minutes, the length of hospital stay was 4 (3–10) days, and all the patients left the ICU on the morning after the procedure. Two patients experienced no postoperative complications, whereas 91% of the complications were minor G1/G2 events. Preliminary assessment also suggested no impairment of the patient’s quality of life.

**Conclusion:**

Our comprehensive protocol might represent a promising all-in-one approach for advanced ovarian cancer. The patient recruitment for this trial is ongoing.

## Introduction

Ovarian cancer is a peritoneal disease, and most patients will ultimately die of tumor progression in the natural history of this gynecologic malignancy. The disease tends to disseminate early into the peritoneal cavity and often remains confined to the peritoneum, which is also the preferred site of recurrence. In these settings, the treatment of the peritoneal cavity has been considered an important point for making a difference in the outcome of ovarian cancer patients, and several studies have assessed the role of intraperitoneal (i.p.) chemotherapy in debulking surgery.^1^–^3^ Usually delivered through a catheter directly into the abdominal cavity, i.p. chemotherapy has demonstrated survival advantages over intravenous (i.v.) chemotherapy that extends beyond 10 years of follow-up.^1^ However, this approach has not been widely accepted in clinical practice mainly due to its higher toxicity, inconvenience, and catheter-related complications,^2^,^4^ as well as the impairment of the patient’s quality of life (QoL) when compared with patients receiving conventional i.v. chemotherapy alone.^5^

Recently, hyperthermic intraperitoneal chemotherapy (HIPEC) has emerged as a main comprehensive treatment of malignancies on the peritoneal surface in association with advanced cytoreductive surgery (CRS), and thus has been considered a promising treatment option for advanced or recurrent ovarian cancer.^6^–^13^ The rationale for using HIPEC is based on the direct cytotoxicity of hyperthermia for malignant cells, the enhancement of this cytotoxicity by anticancer drugs, and the pharmacokinetic advantages of the i.p. route for chemotherapy.^12^ Some studies have also revealed that hyperthermia can reduce the mechanisms of cellular resistance to platins^7^,^8^,^14^ and induce an efficient anticancer immune response via exposure to cell surface heat shock proteins.^15^,^16^ This technique is delivered intraoperatively, avoiding the need for implantation of peritoneal access devices and thereby reducing catheter-related morbidity and tolerance issues.^17^ Despite the potential advantages of HIPEC, there is no clear evidence from randomized controlled trials to advocate this approach as a standard therapy for patients suffering from ovarian cancer. The absence of these solid evidences supports a lot of criticism directed at the increasing use of HIPEC outside of clinical trials.^18^–^23^

Following the skepticism surrounding HIPEC in ovarian cancer, we considered it important to present early outcomes after the interim analysis of a pioneering clinical trial in Brazil. This trial explores the safety and efficacy of a short course of the HIPEC protocol in patients from the Brazilian public health system (i.e., Sistema Único de Saúde [SUS]) under the hypothesis of low morbidity and improved progression-free survival (PFS). Some insights regarding our experience with CRS/HIPEC procedures are also discussed.

## Patients and methods

### Study design and population

A cross-sectional study (interim analysis) was carried out on the women enrolled in our ongoing Phase II trial. This trial was an open-label, double-center, single-arm clinical trial exploring safety and efficacy of neoadjuvant chemotherapy (NACT) followed by CRS plus short-course HIPEC as a comprehensive treatment for patients suffering from advanced epithelial ovarian cancer (EOC). A fast-track recovery strategy was also applied to improve patient outcomes. This trial was conducted under the hypothesis of low morbidity and improved PFS for this all-in-one treatment, and recruited patients from the Brazilian public health system (i.e., SUS) in Pernambuco State since February 2015. The primary end point for this trial is PFS, and the secondary end points are morbidity/mortality, patient-reported QoL, time of restarting systemic chemotherapy after CRS/HIPEC, the length of the ICU and hospital stay, and the overall survival (OS). Calculation of the sample size was based on our preliminary hypothesis that the expected 12-month PFS previously reported with the use of NACT alone^24^,^25^ could be doubled by our comprehensive management involving the HIPEC procedure.^9^,^26^ With both accrual time and a minimum followup period of 2 years, 20 patients were required for analysis considering a one-sided type I error rate of 0.05 and a power of 80%. For safety monitoring, an interim analysis was also planned after completing the predefined trigger of recruiting 50% of patients.

The eligibility criteria for patients for inclusion in the study were that the patients were fit for major surgery and chemotherapy as well as having a biopsy-proven diagnosis of EOC with a clinical stage of IIIB–IV (abdominal only). Additionally, the patients need to be aged 18–70 years, have a performance status of 0–2 (Eastern Cooperative Oncology Group) and/or >70 points on the Karnofsky scale, and should have signed the consent form. We excluded patients who showed evidence of extensive retroperitoneal lymph node involvement or unresectable disease (i.e., massive involvement of the small bowel, mesentery, or hepatic pedicle, and ureteral or biliary obstruction), as well as disease progression, infection, or health impairment during NACT; limiting visceral obesity for surgical purposes; and residual disease after CRS that was ≥2.5 mm (i.e., CC-2 and CC-3).

The study protocol was approved by the Ethics Research Committees of Instituto de Medicina Integral Professor Fernando Figueira (IMIP) and the Brazilian National Ethics Research Committees – CONEP (CAAE: 18388113. 4.0000.5201), and registered on ClinicalTrials.gov under the identifier NCT02249013. Written informed consent was obtained from all patients, and the procedures complied with the standards set by the Declaration of Helsinki and the current Brazilian ethical guidelines.

### Variables and outcomes

Clinical data on the patients enrolled in our trial were prospectively assessed and recorded by electronic spreadsheets. Follow-up scheduling for patient monitoring included clinical pelvic/general examination, and assessment of CA125 every 3 months for 2 years, every 6 months for the next 3 years, and then, annually. Imaging exams were also performed every 6–12 months or when clinically required, for at least 2 years and annually, thereafter.

Response to chemotherapy and progression were defined according to the Response Evaluation Criteria in Solid Tumors (RECIST) and the Gynecologic Cancer Intergroup (GCIG) criteria. We defined PFS as the time from the start of NACT until the date of first progression or death and the OS as the time until death; however, the data on patient survival were not explored at the time of this interim analysis. We measured the QoL using the European Organisation for Research and Treatment of Cancer (EORTC) questionnaire QLQ-C30 v.3.0. This health-related questionnaire was completed at baseline just before the CRS/HIPEC procedure (i.e., at the time of hospital admission), after the CRS/HIPEC (i.e., at the time of restarting the systemic chemotherapy), and after completion of the entire protocol (i.e., at 3–6 weeks after the last systemic chemotherapy cycle). The scales and items of the questionnaire were linearly transformed and analyzed according to the EORTC QoL group procedures.

For descriptive analyses, we summarized the continuous variables as medians (interquartile range) and categorical variables as frequencies (percent). Statistical analyses were not necessary for this interim analysis, and charts were created using Microsoft^®^ Office for Mac 2011 (v.14.2.1).

### Treatment protocol

At screening, all women received a comprehensive assessment of the risk factors for suboptimal cytoreduction based on clinical, radiological, and surgical findings (i.e., previous laparotomy or staging laparoscopy/laparotomy), as well as the concentrations of serum tumor markers.^27^–^29^ Patients with a high tumor burden were then assigned to receive 2–4 cycles of NACT followed by fast-track CRS, plus a short course of HIPEC for all patients who had a response or stable disease, which was then followed by 2–4 cycles of postoperative chemotherapy. Systemic chemotherapy was scheduled in a total of 6 cycles of the standard combination of carboplatin (AUC 6) and paclitaxel (175 mg/m^2^) administered every 21 days, adopting the usual criteria for dose modification or delay, as appropriate.

Standard CRS comprises total abdominal hysterectomy, bilateral salpingo-oophorectomy, omentectomy, and maximum debulking of metastatic tumors. Systematic pelvic and/or aortic lymphadenectomy was performed at the surgeon’s discretion in patients with clinically suspicious nodal involve ment. Whenever needed, advanced CRS procedures also involved parietal peritonectomies and visceral resections, as previously standardized.^30^ However, a more conservative policy using high-voltage electrocoagulation, traditional scissors or knife resections, and other minor procedures was adopted as much as possible to reduce morbidity, confining complete peritonectomy to where there is evidence of a more bulky or confluent disease.

HIPEC was performed according to the closed-abdomen technique, using cisplatin (25 mg/L of perfusate/m^2^, total limit of 240 mg) for 30 minutes, with an intra-abdominal target temperature of 41°C–43°C. The perfusate (2 L/m^2^, ranging from a minimum of 4 L to a maximum of 6 L) was circulated using an extracorporeal circulation device called the Performer HT (RanD, Medolla, Italy) at a flow rate of 700 mL/min. This HIPEC protocol was named “short course” based on its 30-minute length.

### Fast-track recovery strategy

A comprehensive fast-track program was planned to accelerate recovery, reduce morbidity, and shorten convalescence for patients enrolled in our trial. All patients were admitted 1 day before surgery. A soft diet was permitted until late at night, and a chlorhexidine shower was recommended. We do not routinely recommend the systematic mechanical preparation of the colon, but patients with a previous history of constipation were provided with a single dose (500 mL) of a 12% glycerin solution administered rectally for bowel preparation.

Anesthetic management included the positioning of a low thoracic epidural catheter in association with the inhalational and i.v. general anesthesia and strict monitoring to maintain the temperature and i.v. fluid needs of the patient. The fluid therapy regimen was used to maintain a mean arterial pressure ≥65–75 mmHg, a central venous pressure from 8 to 12 mmHg, and central venous oxygen saturation ≥70%. The patients were transfused with a concentrated red cells with Hb values <8 mg/dL. The empiric use of prophylaxis antibiotics (i.e., ampicillin/sulbactam) was initiated at the time of operation and continued postoperatively for 24 hours. The preemptive prophylaxis of postoperative nausea and vomiting included administration of metoclopramide (10 mg, 1 hour before surgery), dexamethasone (10 mg, at the time of induction of anesthesia), and ondansetron (8 mg, immediately after surgery). During the HIPEC phase, fresh-frozen plasma was administered (1 U/15 min), and diuresis was maintained at values ≥120 mL/15 min by optimization of the hemodynamic parameters and/or using a low dose of diuretics (i.e., furosemide), as appropriate. At this period, we also started an i.v. infusion of 10% MgSO_4_ (2 g over 2 hours, startinĝ1 hour before HIPEC) to prevent cisplatin-induced nephrotoxicity. Abdominal drains and colostomies were avoided as much as possible, and the nasogastric tube was removed after the intervention. Following surgery, patients were extubated in the operating room when possible and were transferred to the ICU.

Postoperative treatments included analgesia using epidural and venous nonopioid drugs and i.v. drip therapy adjusted according to individual needs. Venous thrombo-embolism prophylaxis with low-molecular-weight heparin was only administered after 12–24 hours when its safety was confirmed by laboratory test. Urinary catheters were removed on the first postoperative day unless contraindicated, and the patients required early mobilization out of bed. Early oral feeding was also introduced on the first day, and bowel stimulation with 30 mL/day of oral magnesium hydroxide and prokinetics (i.e., metoclopramide 10 mg q8 h, i.v.) was applied for 48 hours (or presence of flatus) to prevent postoperative paralytic ileus. Criteria for hospital discharge included tolerance to regular diet and satisfactory pain control with oral agents alone.

## Results

**Figure 1:**
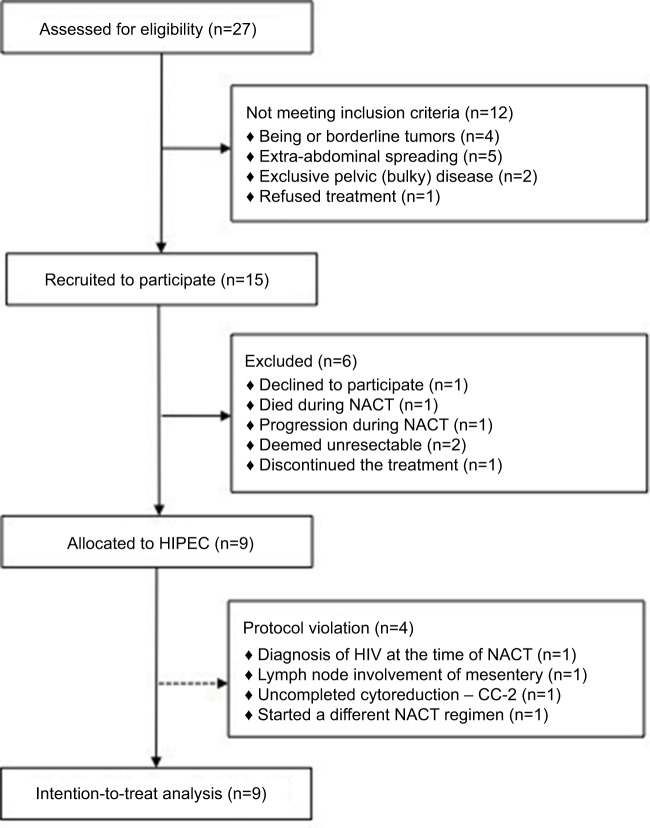
Flow diagram summarizing the number of patients who were assessed for eligibility, recruited to participate, assigned to HIPEC, and included in the analyses. **Abbreviations:** HIPEC, hyperthermic intraperitoneal chemotherapy; NACT, neoadjuvant chemotherapy.

Twenty-seven patients were screened for eligibility, and finally, nine patients with stage III EOC were allocated to the HIPEC procedure from February 2015 to July 2017. These include four patients who met some exclusion criteria but ultimately underwent HIPEC, as shown in the flow diagram (Figure 1). Because of slow accrual, the planned interim analysis was anticipated, and the patient’s data were explored according to the intention-to-treat principle focusing on the HIPEC procedures. The baseline demographic and preoperative clinical characteristics of the enrolled patients are presented in Table 1. The median (range) CA125 serum levels at diagnosis, after NACT, after CRS/HIPEC, and at the end of protocol were 692 (223.7–655.0), 35.7 (18.5–374.6), 34 (11.6–146.5), and 14.2 (7.8–57.8) U/mL, respectively. All the patients completed a total of 6 cycles of perioperative i.v. chemotherapy, as planned, in association with CRS/HIPEC.

The same surgical team performed all CRS/HIPEC procedures in the same participating hospital (i.e., IMIP). Systematic lymphadenectomies were not routinely performed in five of the nine patients, while four underwent para-aortic lymph node dissection with (n=2) or without (n=2) pelvic lymphadenectomy. As part of the CRS, four patients required bowel resection, such as rectosigmoidectomy (n=3) or partial colectomy (n=1), but no ostomies were performed and only one patient received pelvic drainage. All patients left the ICU on the morning after the procedure, whereas about 91% of postoperative complications were minor G1/G2 complications, according to the Clavien–Dindo classification. The most common morbidities were minor G1/G2 vomiting (n=2) and G3 anemia (n=2), according to the National Cancer Institute Common Terminology Criteria for Adverse Events (NCI/CTCAE) classification version 4.0. Only one patient experienced reoperation at the fourth postoperative day because of G3 postoperative hemorrhage, but no deaths or long-term complications were recorded. Tables 2 and 3 summarize most of the operative characteristics and the postoperative complication rates.

**Figure 2:**
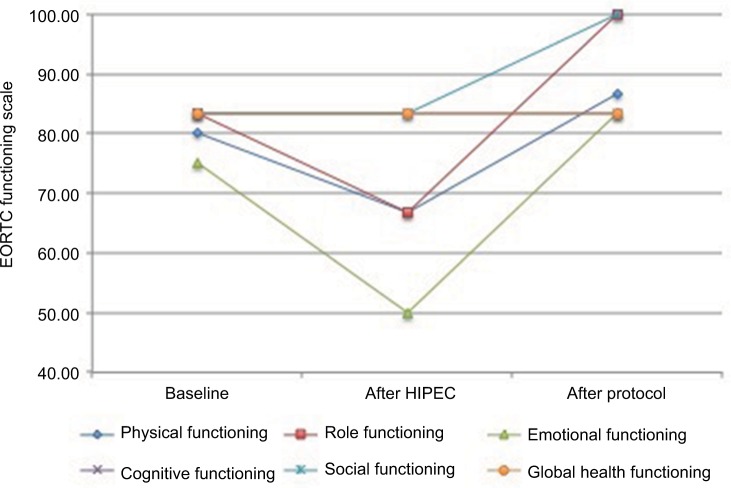
Course of the patient-reported health-related quality of life over time, according to EORTC QLQ-C30 functioning scales. All subscale responses were converted to 0–100 scales (according to the EORTC guidelines). **Abbreviations:** EORTC, European Organisation for Research and Treatment of Cancer; HIPEC, hyperthermic intraperitoneal chemotherapy.

A baseline EORTC QLQ-C30 questionnaire and at least one follow-up questionnaire were received from all the patients. Seven of the nine patients completed follow-up questionnaires “after HIPEC”, and five completed “after protocol”. The preliminary data on the QoL of the patients were assessed only as functioning scales and are summarized in Figure 2.

## Discussion

Upfront CRS followed by platinum-based chemotherapy is the mainstay of treatment for advanced disease and has been our preferred multimodal treatment for patients eligible for the oncologic surgical procedure. However, ovarian cancer is often diagnosed at a later stage and in elderly patients who are then referred to specialists at a high perioperative risk profile or a low likelihood of achieving cytoreduction, especially in the context of the SUS public health system. In these settings, NACT may offer rapid symptomatic improvement and reduction in tumor burden, which helps in the selection and preparation of patients for aggressive treatment options, such as advanced CRS. This approach may also contribute to reducing the invasiveness of treatment and perioperative morbidity with noninferior outcomes with respect to PFS and OS.^24^,^25^,^31^–^33^ Some pieces of evidence also suggested the effectiveness of NACT followed by interval debulking surgery and i.p. chemotherapy (delivered by means of abdominal catheters).^34^,^35^ We thus considered NACT as an important component for our study protocol involving HIPEC.

Despite the established rationale and encouraging results favoring the use of NACT, this approach has also been related to a higher risk of developing platinum resistance.^36^ Accordingly, we reinforced the concept of early and complete removal of all macroscopic tumors in the therapeutic sequence of EOC, and thus, we limited NACT to 2–4 cycles before surgery with the intention of minimizing the risk of chemoresistance.^37^ Our protocol additionally adopted a more flexible policy regarding the number of preoperative cycles of chemotherapy to allow for a more individualized decision in terms of the best moment to proceed with the CRS/HIPEC procedures, which accounts for a balance of variables such as the improvement of health status, tumor response (i.e., CA125 response by GCIG and at least stable disease according to RECIST), and operating room scheduling. At this point, HIPEC also appears complementary to NACT in reducing the mechanisms of cellular resistance to platins,^7^,^8^,^14^ while some clinical studies revealed its protective value against chemoresistance.^7^,^8^

Recent literature has supported the hypothesis of improvement in the survival associated with HIPEC for advanced and recurrent ovarian cancer.^6^–^9^,^13^,^38^,^39^ For example, Spiliotis et al^7^ presented a pioneering Phase III trial exploring the use of HIPEC for recurrent disease and demonstrated a survival advantage favoring the use of HIPEC. A main interesting finding of this study was the similar rate of survival observed in both the platinum-sensitive and platinum-resistant subgroups, which is in line with the previous report by the FROGHI (French Oncologic and Gynecologic HIPEC) group of a multicenter retrospective cohort study of 474 patients with recurrent EOC.^8^ Despite the merit of this pioneering study, it has been criticized because of the many drawbacks in its presentation and methods.^19^,^21^ The role of HIPEC in advanced EOC was also explored in three European Phase II trials. In the study conducted by Deraco et al involving upfront CRS/HIPEC,^9^ all the patients, except one who died postoperatively, started adjuvant systemic chemotherapy after a median of 46 (29–75) days, which represents a relative delay compared to our results (37 [33–50] days). In the strategy adopted by Gouy et al^38^ combining 6 cycles of NACT, CRS/HIPEC, and postoperative maintenance bevacizumab, the median interval between the last cycle of NACT and the CRS/HIPEC was 41 (24–81) days, which contrasts with our better results (29 [26–43] days) in these settings. In the study by D’Hondt et al exploring interval CRS plus HIPEC after 3–4 cycles of NACT,^26^ the time to starting the adjuvant systemic chemotherapy was 42 (14–89) days. In all these trials, the addition of HIPEC seemed to be a promising strategy for the treatment of advanced EOC in terms of survival, whereas our approach initially suggested some advantages favoring toxicity and postoperative outcomes, especially the length of the hospital stay – our postoperative hospital stay was only 4 (3–10) days, compared to 21 (13–67), 18.5 (10–69), and 15 (10–69) days in the cited trials. Accordingly, our approach could be presented as a promising all-in-one approach if some survival advantage could be confirmed in the final analysis, including survival outcomes for this trial.

More recently, Van Driel et al^39^ and Lim et al^40^ presented preliminary data from their Phase III trials (NCT00426257 and NCT01091636, respectively). In the former study, patients who showed at least stable disease after three cycles of NACT, and who had no residual mass greater than 2.5 mm, were randomly assigned to receive intervals of CRS with or without HIPEC using cisplatin (100 mg/m^2^) for 90 minutes. Three additional cycles of i.v. chemotherapy were also given postoperatively. The time of restarting chemotherapy was 33 days, with a hospital stay of 10 days. HIPEC was associated with a longer recurrence-free survival and a significant improvement in the OS (48 vs. 34 months; HR, 0.64; 95% CI, 0.45–0.91; *P*=0.01), whereas the number of patients with G3/G4 adverse events was also similar in both treatment arms (28% vs. 24%; *P*=0.61). In the second trial, the HIPEC regimen comprised cisplatin at the dose of 75 mg/m^2^ for 90 minutes and NACT was allowed, but not systematically applied. The eligibility criteria for intraoperative randomization were based on residual tumors <1 cm. With this study design, the authors found no statistical superiority for HIPEC in terms of the survival, but the subgroup of women who received NACT showed a gradual distinction trend favoring the HIPEC group, where the 5-year OS was 47.9% in the HIPEC arm and 27.7% in the control arm. In summary, these early results highlight the clinical importance of combining HIPEC with NACT, including the role of HIPEC for patients with residual tumors no greater than 2.5 mm. This is probably linked to the potential effect of hyperthermia in modifying factors of cancer growth, the microenvironment, immune response, vascularization, and oxygen supply that could serve to improve the outcomes in ovarian cancer.^41^

Despite the fact that CRS/HIPEC practices are widely variable,^12^,^42^ the majority of HIPEC studies on ovarian cancer have used i.p. cisplatin,^8^,^12^,^42^,^43^ which could also be employed in routine clinical practice as a single agent according to most experts.^42^ The duration of perfusion with this drug may reach 160 minutes (usually ranging from 30 to 120 minutes) in line with the investigator’s experience and the protocol to be used,^8^,^12^,^42^ but consequently, a higher procedure length may also imply major morbidity.^43^ Additionally, some data have supported an increased drug concentration in the instillation with a shorter bathing duration would probably give similar pharmacokinetic results to those with a longer bathing duration and decreased drug concentration.^44^,^45^ In these settings, we proposed a short-course (i.e., 30 minutes), high-dose (i.e., 25 mg/m^2^/L) cisplatin schedule as the drug protocol for our study, supposing that it could be a low-morbidity but equally effective regimen to be applied to our comprehensive approach. At the time of this interim safety analysis, the lower morbidity of this regimen can be based on our low rates of complication and short length of hospital stay.

Our study was limited by the slow accrual, which led us to anticipate this interim analysis and to work inviting other Brazilian cancer centers to participate in this trial. With these efforts, we hope to complete our targeted accrual in the following years, while waiting for the results of many ongoing trials addressing the issue of HIPEC in ovarian cancer. Another point is that the study protocol lacks the ability to provide routine laparoscopic estimation of tumor burden at diagnosis for all our patients, as previously published.^33^ Because of our initial interest in including patients who were referred to our tertiary-care centers after some surgical exploration by a general gynecologist, the selection of patients with a low likelihood of achieving an upfront complete cytoreduction was planned based on comprehensive evaluation of the clinical status, serum CA125 levels, CT scan findings,^27^,^28^ and reports of the first exploratory surgery, whereas an initial staging laparotomy or laparoscopy was not performed by our team in one of the nine cases. Accordingly, only one patient with Federation of Gynecology and Obstetrics (FIGO) stage IIIB at laparotomy staging was recruited due to disease spreading into the upper abdomen and diffusing in the mesentery, while all other patients were considered as having bulky stage IIIC disease. Since the tumor load remains an independent and poor prognostic factor despite the completeness of cytoreduction,^46^,^47^ we sincerely believe that preoperative measurement plays a role in clinical trials exploring new strategies for advanced EOC patients. Additional criticisms of our protocol might involve the lack of baseline QoL measurements just before starting NACT because our focus in this sub-analysis was directed at the CRS/HIPEC component of our protocol. On the other hand, the strengths of this study include the fact that it is a former clinical trial involving HIPEC procedures in Brazil and the first trial to use the Performer HT device (RanD). This includes efforts for conducting this kind of study in the context of the public health system from a developing country, and finally, the pioneering exploration of a comprehensive strategy combining perioperative chemotherapy (i.e., NACT plus adjuvant chemotherapy), advanced CRS, fast-track recovery procedures, and a short-course HIPEC for advanced EOC.

## Conclusion

Because most of the criticism surrounding the use of HIPEC in ovarian cancer involves the inherent potential morbidity of this approach,^48^ we considered it important to present an interim analysis of our trial that suggests the low morbidity and lack of impairment of the patient’s QoL with the adoption of comprehensive treatment involving HIPEC. Although this current paper does not yet focus on the efficacy of HIPEC (data about PFS and OS are not matured and recruitment is ongoing), the issue could be potentially interesting. In our opinion, this is a promising approach that should be evaluated in the management of EOC, especially when other combined i.p. chemotherapy regimens and sophisticated target therapies failed to demonstrate an advantage for patients with advanced disease.^3^ Herein, our all-in-one protocol seems to be feasible, safe, and simple for the patient, surgeon, and nursing caregivers. It has advantages in combining the early i.p. route of chemotherapy without the need for abdominal catheters, the synergism of hyperthermia, and the benefits of NACT and the fast-track recovery procedures allowing for earlier patient mobility, recovery, and hospital discharge.

## Figures and Tables

**Table 1:** Baseline demographic and preoperative clinical characteristics

Variable	Median (range) or n (%)
Age (years)	46 (19–63)
Body mass index	21.5 (16.5–29.1)
Performance status (ECOG)^a^	
0	1 (11.1)
1	6 (66.7)
2	2 (22.2)
ASA classification	
I	4 (44.4)
II	5 (55.6)
Charlson comorbidity index	
0–2	2 (22.2)
3–5	5 (55.6)
>5	2 (22.2)
Prior surgical score	
0	4 (44.4)
1	4 (44.4)
2	1 (11.2)
FIGO staging	
IIIB	1 (11.1)
IIIC	8 (88.9)
Histology (WHO)	
High-grade serous	6 (66.7)
Endometrioid	1 (11.1)
Mixed epithelial	1 (11.1)
Serum CA125 (U/mL) at diagnosis	692 (223.7–6550)
Number of cycles of neoadjuvant chemotherapy	3 (2–4)
Number of cycles of adjuvant chemotherapy	3 (2–4)

**Note:**

a Performance status at the time of CRS/HIPEC (after NACT).

**Abbreviations:** ASA, American Society of Anesthesiologists; CRS, cytoreductive surgery; ECOG, Eastern Cooperative Oncology Group; FIGO, International Federation of Gynecology and Obstetrics; HIPEC, hyperthermic intraperitoneal chemotherapy; NACT, neoadjuvant chemotherapy; WHO, World Health Organization.

**Table 2:** Operative and postoperative clinical characteristics

Variables	Median (range) or n (%)
Peritoneal cancer index	9 (3–18)
Completeness of cytoreduction	
CC-0	8 (88.9)
CC-2	1 (11.1)
Operative time (minutes)	395 (235–760)
Time of perfusion^a^ (minutes)	50 (43–58)
Mean temperature (°C)	42.1 (41.2–42.5)
Chemotherapy dose (mg)	170 (140–220)
Hospital stay (days)	4 (3–10)
Time to CRS/HIPEC after NACT (days)	29 (26–43)
Time to chemo after HIPEC (days)	37 (33–50)

**Notes:**

a Total time after the “patient-filling phase”, while waiting for stable temperatures. The “drug circulation phase” (i.e., HIPEC) was 30 minutes in all cases.

**Abbreviations:** CRS, cytoreductive surgery; HIPEC, hyperthermic intraperitoneal chemotherapy; NACT, neoadjuvant chemotherapy.

**Table 3:** Postoperative complication rates^a^

Variables	Median (range) or n (%)
Number of complications^b^ (per patient)	1 (0–3)
Patients with no complications (G0)	2 (22.2)
Minor complications (G1/G2)	
Vomiting^c^	2 (22.2)
Abdominal distension (G1)	1 (11.1)
Wound infection (G2)	1 (11.1)
Catheter-related infection (G2)	1 (11.1)
Hypokalemia (G1)	1 (11.1)
Lymphocele (G1)	1 (11.1)
Major complications (G3/G4)	
Anemia (G3)	2 (22.2)
Vomiting (G3)	1 (11.1)
Postoperative hemorrhage (G3)	1 (11.1)
Postoperative death (G5)	0 (0)

**Notes:**

a According to both the National Cancer Institute Common Terminology Criteria for Adverse Events (NCI/CTCAE), version 4.0.

b A total of 11 complications were recorded.

c One case was ranked as G1, and the other one as G2.
